# Search methods for prognostic factor systematic reviews: a methodologic investigation

**DOI:** 10.5195/jmla.2021.939

**Published:** 2021-01-01

**Authors:** Leah Boulos, Rachel Ogilvie, Jill A. Hayden

**Affiliations:** 1 LeahM.Boulos@nshealth.ca, Evidence Synthesis Coordinator, Maritime SPOR SUPPORT Unit, Halifax, NS, Canada; 2 Rachel.Ogilvie@dal.ca, Research Program Coordinator, Department of Community Health and Epidemiology, Dalhousie University, Halifax, NS, Canada; 3 JHayden@dal.ca, Associate Professor, Department of Community Health and Epidemiology, Dalhousie University, Halifax, NS, Canada

## Abstract

**Objective:**

This study retroactively investigated the search used in a 2019 review by Hayden et al., one of the first systematic reviews of prognostic factors that was published in the Cochrane Library. The review was designed to address recognized weaknesses in reviews of prognosis by using multiple supplementary search methods in addition to traditional electronic database searching.

**Methods:**

The authors used four approaches to comprehensively assess aspects of systematic review literature searching for prognostic factor studies: (1) comparison of search recall of broad versus focused electronic search strategies, (2) linking of search methods of origin for eligible studies, (3) analysis of impact of supplementary search methods on meta-analysis conclusions, and (4) analysis of prognosis filter performance.

**Results:**

The review's focused electronic search strategy resulted in a 91% reduction in recall, compared to a broader version. Had the team relied on the focused search strategy without using supplementary search methods, they would have missed 23 of 58 eligible studies that were indexed in MEDLINE; additionally, the number of included studies in 2 of the review's primary outcome meta-analyses would have changed. Using a broader strategy without supplementary searches would still have missed 5 studies. The prognosis filter used in the review demonstrated the highest sensitivity of any of the filters tested.

**Conclusions:**

Our study results support recommendations for supplementary search methods made by prominent systematic review methodologists. Leaving out any supplemental search methods would have resulted in missed studies, and these omissions would not have been prevented by using a broader search strategy or any of the other prognosis filters tested.

**Figure:**
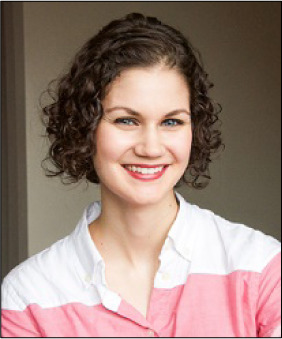
Leah Boulos

## INTRODUCTION

Systematic reviews of prognosis studies are being published at an increasing rate. Such reviews typically address one or more of the following aims: identify the most likely course for a specific condition (overall prognosis) [1]; identify what characteristics are associated with, or predict, a given outcome (prognostic factors) [2]; identify cohorts with specific characteristics who are more or less likely to experience a given outcome (predictive models) [3]; and/or identify the characteristics or factors that impact the effectiveness of a specific treatment (treatment effect modification) [4]. Each of these aims synthesizes different aspects of prognosis or clinical prediction studies, and therefore, each requires distinct search approaches.

Prognosis studies can be difficult to retrieve in traditional searches of electronic databases [5]. Although several search filters have been developed and validated to identify prognosis studies [6–9], the sensitivity of these filters tends to be low [9, 10]. There is also a lack of consensus on the best approach to search for prognosis studies [5, 10]. This is in large part due to the variability in language that is used to describe prognosis in research articles. Without consistent prognosis terminology, it is difficult for filter developers to determine the optimal combination of search terms. Prognosis-related controlled vocabulary tends to be quite broad, and prognosis studies are often poorly and/or inconsistently indexed [5].

Prognostic factor studies pose additional challenges for searchers. They present similar issues with inconsistent terminology, and the addition of search terms that are related to the prognostic factors of interest can significantly reduce retrieval while also reducing sensitivity, increasing the likelihood of missing relevant studies. Riley et al. published a guide to systematic review and meta-analysis of prognostic factor studies in 2019 [11], but its guidance on searching was specific to clinical prediction studies, rather than prognostic factor studies or prognosis studies in general. Searches for clinical prediction studies require their own unique language that can be found in existing search filters [12–16]. This leaves a gap in the understanding of best practices for searching for prognostic factor studies and prognosis studies in general.

Greenhalgh and Peacock found that “protocol-driven” database searches and hand searches alone resulted in only 30% of sources being found in a systematic review of complex evidence [17]. Later, in an overview of 17 reviews of low back pain prognosis, Hayden et al. found many methodological shortcomings in the reviews that they studied, including inadequate search approaches [18]. These findings were corroborated in a subsequent Cochrane review protocol by Hayden et al. [19], which reported that of the more than 35 prospective cohort studies that were likely to be included in their review, less than 30% were included in 3 existing systematic reviews on the same topic [20–22]. Furthermore, those existing reviews included only a small number of overlapping studies, suggesting that each employed different approaches to searching.

The authors posit that traditional, highly sensitive searches of electronic databases are inadequate to systematically retrieve prognosis studies and that combining a more focused electronic database search with supplementary search methods can be a more effective approach. We define supplementary search methods as any means of locating studies other than query-based database searching. This most often includes hand searching, reference searching and citation tracking (sometimes called backward and forward searching), liaising with subject area experts, and consulting researchers' personal files. These methods are demonstrably effective approaches in several case examples [17, 23–26] and are recommended in the searching chapter of the Cochrane Handbook [27]. This approach harnesses the human capacity for evaluation and judgment, which can be difficult to communicate through traditional electronic database search language—especially when search concepts are difficult to define and use inconsistent terminology. Additionally, by extending beyond the scope of a database search alone, supplementary search methods help mitigate the risk of bias introduced by using prognostic factor terms to focus an electronic search [28].

To test our hypothesis and make recommendations for future practice, we conducted a methodologic investigation of Hayden et al.'s review on the association between recovery expectations and disability outcomes in adults with low back pain [28]. This review is one of the first systematic reviews of prognostic factor studies in the Cochrane Library. It is notable for featuring a novel search strategy that combined a focused electronic database search with extensive supplementary search methods. Our objective was to retrospectively investigate and assess the search methods used in Hayden et al.'s review, highlighting experiences and lessons learned in order to provide guidance for future reviewers of prognostic factor studies.

## METHODS

Using data from Hayden et al.'s example review [28], we used four approaches to comprehensively assess aspects of systematic searching for prognostic factor studies: (1) comparison of search recall of broad versus focused electronic search strategies, (2) linking of search methods of origin for eligible studies, (3) analysis of impact of supplementary search methods on meta-analysis conclusions, and (4) analysis of prognosis filter performance.

### Comparison of search recall of broad versus focused electronic search strategies

The first component of Hayden et al.'s search strategy was a focused electronic search for prognostic factor studies (supplemental Appendix A) that combined search terms for low back pain, a prognosis filter, and terms related to the prognostic factor of interest, in this case, recovery expectations. To assess the feasibility of running a broad electronic search versus a focused search combined with supplementary methods, in November 2019, we used Ovid MEDLINE to rerun Hayden et al.'s focused search (low back pain AND prognosis filter AND recovery expectations) and compare its recall with that of a broader search without recovery expectations terms (low back pain AND prognosis filter).

### Linking of search methods of origin for eligible studies

In addition to database searching, Hayden et al. used multiple supplementary search methods: a reference search of previously published systematic reviews, which were retrieved using a broader electronic search that did not include the prognostic factor concept; a forward search of publications citing identified prognostic factor measures; and hand searching, reference searching of included studies, and consultation of personal files to identify additional studies. To determine which studies would have been missed had any of the search strategy components been left out, we linked each included study in Hayden et al. to its search methods of origin, focusing only on those studies indexed in Ovid MEDLINE (n=58 of 60 total included studies).

To confirm which studies were retrieved by the focused electronic search strategy, we created a search string comprising the PubMed identifiers (PMIDs) of each of the fifty-eight included MEDLINE-indexed studies and combined it with the search that the team used. We consulted the team's search records to determine which studies had been retrieved by supplementary search methods. Finally, in addition to tracing the origin of each included study back to the search methods used in the review, we explored a hypothetical scenario in which only the broad electronic search strategy was used.

### Analysis of impact of supplementary search methods on meta-analysis conclusions

Hayden et al.'s review included unadjusted and adjusted meta-analyses (MAs) of the effect of recovery expectations on four outcomes: work participation, important recovery outcomes, functional limitations, and pain intensity. To gauge the impact of supplementary search methods on the MAs' conclusions, we repeated them with only those studies retrieved by the focused electronic search strategy. We then ran a similar experiment to see if the hypothetical broader search would have made any difference to the MAs' conclusions.

### Analysis of prognosis filter performance

We conducted a performance analysis of the prognosis filter used in Hayden et al.'s review (Irvin filter) [29] compared with three other known filters: the optimized version of a filter developed by the Hedges Team at McMaster University (“Hedges Optimized” filter) [9]; an inclusive, general filter developed by Parker et al. (“Inclusive General” filter) [30]; and another filter developed by Parker et al. combining all of the Hedges Team's filters [9] plus the keywords “natural history” (“Combined Hedges + Natural History” filter) [30]. Table 1 shows the details of all filters we analyzed in Ovid MEDLINE format. Although the four filters contain similar terms, they demonstrate the slight variance in language that is often used when searching for prognosis studies. Through our analysis, we explored whether these minute differences affected recall.

**Table 1:** Details of prognosis filters analyzed, Ovid MEDLINE format

Irvin [29]	Hedges Optimized [9]	Inclusive General [30]	Combined Hedges + Natural History [30]
Cohort Studies/ incidence.tw. Mortality/ Follow-Up Studies/ prognos*.tw. predict*.tw. course.tw. Survival Analysis/ or/1-8	prognosis.sh. diagnosed.tw. cohort:.mp. predictor:.tw. death.tw. exp models, statistical/ or/1-6	cohort.ti,ab. incidence.ti,ab. mortality.ti,ab. follow-up study.ti,ab. follow-up studies.ti,ab. prognos*.ti,ab. predict*.ti,ab. course.ti,ab. natural history.ti,ab. or/1-9	Incidence/ exp Mortality/ Follow-Up Studies/ prognos*.tw. predict*.tw. course*.tw. (first and episode).ti,ab. cohort.ti,ab. natural history.tw. or/1-9

The performance analysis measured operating characteristics for each filter against a reference standard of records in Ovid MEDLINE using formulae outlined by Kok et al. [31] and Gehanno et al. [32]. To provide richer ground for analysis, we wanted to expand our reference standard beyond the sixty studies included in the final stage of Hayden et al.'s review. As detailed in the original Hayden et al. review, to mitigate the risk of introducing bias by including prognostic factor terms in the electronic search, the team advanced all citations—retrieved by any of the search methods detailed thus far—of low back pain prognostic factor studies, regardless of the presence or absence of the specific prognostic factor of interest. We, therefore, drew the reference standard from prognosis studies that were included at the first stage of screening in the review. We used the reference standard to calculate each filter's sensitivity, precision, number needed to read (NNR), specificity, and accuracy.

## RESULTS

### Comparison of search recall of broad versus focused electronic search strategies

The broad electronic search strategy (no prognostic factor component) retrieved 15,242 records in MEDLINE (run November 2019). The focused electronic search strategy (prognostic factor—i.e., “expectations”—terms included) retrieved 1,332 records. Including prognostic factor terms resulted in a 91% reduction in the number of retrieved records.

### Linking of search methods of origin for eligible studies

**Figure 1:**
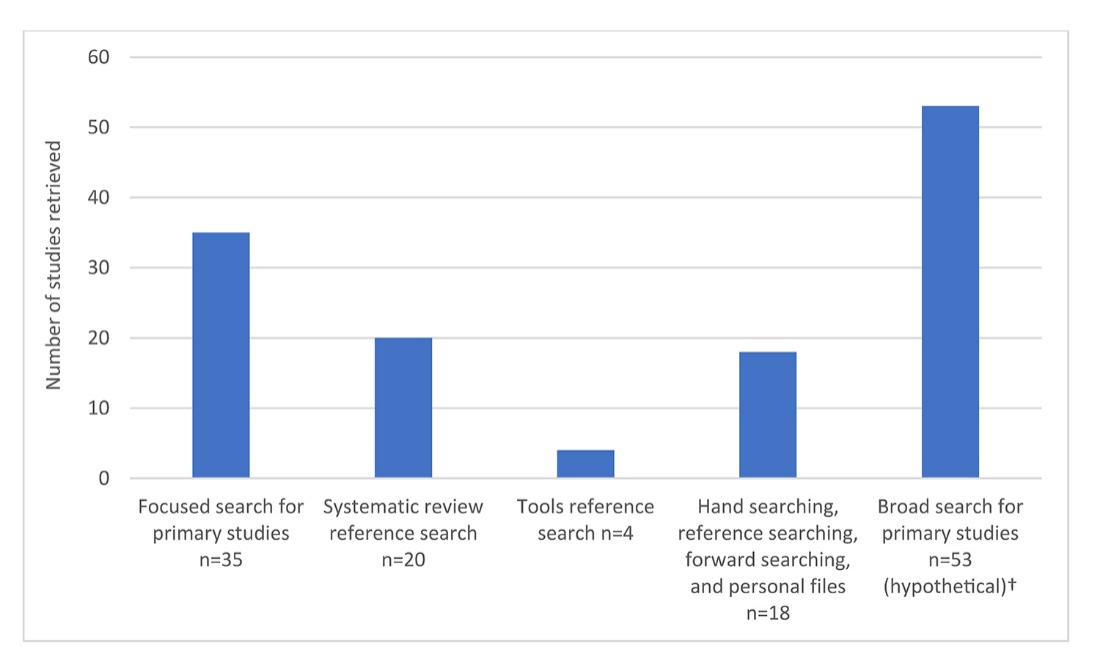
Visualization of studies indexed in Ovid MEDLINE (n=58)* retrieved by each search component used in Hayden et al.'s review [28], including the hypothetical broad electronic search

Figure 1 shows the search methods of origin for each included study indexed in MEDLINE (n=58 of 60 included studies). The numbers in each column do not add up to 100, as some studies were retrieved by multiple methods. The underlying data for this figure are found in supplemental Appendix B.

* Search recall and search filter analyses in this methodologic investigation were limited to Ovid MEDLINE. Incidentally, the two studies not located in Ovid MEDLINE were not included in the review's meta-analyses.

† A broad electronic database search for primary studies was not undertaken as part of the review's actual search methods; this is a hypothetical search to show the difference in recall.

**Figure 2:**
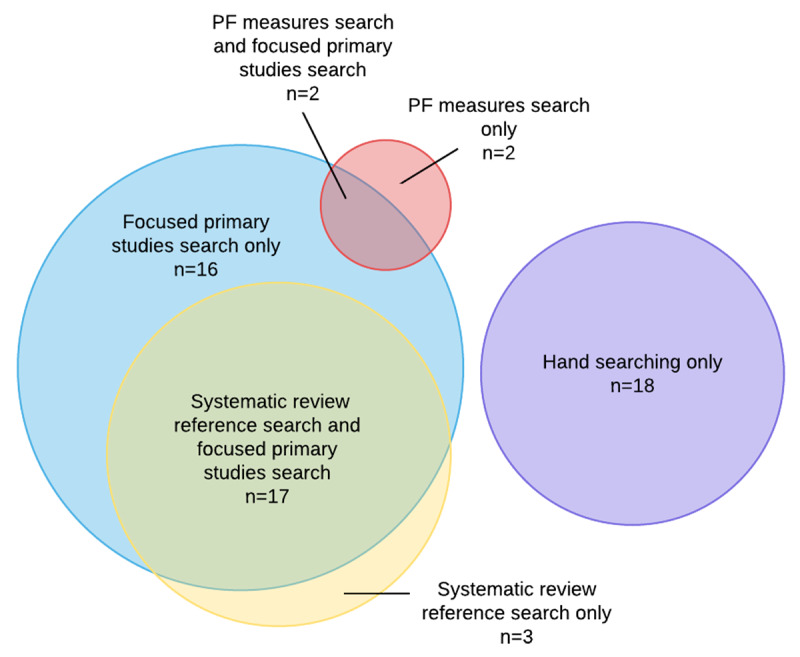
Visualization of overlap in recall of each search component used in Hayden et al.'s review [28]*

Figure 2 visualizes the overlap in search results from each component of the search strategy. The focused electronic database search for primary studies retrieved thirty-five records; the systematic review reference search retrieved twenty; the forward search of publications citing identifying prognostic factor measures retrieved four; and hand searching, reference searching, and consulting personal files retrieved eighteen. There was overlap between some components: seventeen records were retrieved by both of the first two methods, and two were retrieved by both the focused electronic search and the forward search for prognostic factor measures. The portions of the diagram that do not overlap with any other portions represent studies that would not have been found by any other method. Because each method features a portion without any overlap, the diagram demonstrates that none of the methods could have been left out. Most notable of these was hand searching, which did not overlap with any of the other methods, as the purpose and approach of hand searching is to locate studies that are not retrieved by other previous methods.

PF=prognostic factor.

* Figures offer visual estimates for illustrative purposes only and are not mathematically proportional.

**Figure 3:**
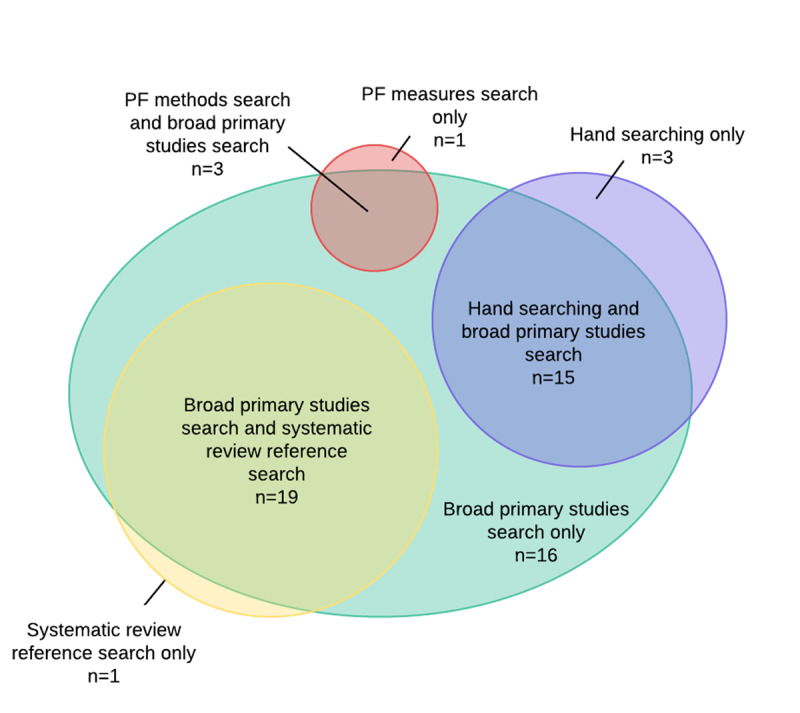
Visualization of overlap in recall of each search component used in Hayden et al.'s review [28], substituting the focused electronic database search for primary studies with a hypothetical broad electronic search*

In the hypothetical scenario in which only a broad electronic search strategy was used, fifty-three of fifty-eight studies indexed in Ovid MEDLINE would have been retrieved. Figure 3 visualizes this hypothetical scenario. Although the portions with overlap are larger than in Figure 2, the portions without overlap demonstrate that even with a broader electronic database search, supplementary search methods would still have been necessary to retrieve all included studies.

* Figures offer visual estimates for illustrative purposes only and are not mathematically proportional.

### Analysis of impact of supplementary search methods on meta-analysis conclusions

Removing studies that were not found by the focused electronic search resulted in changes in two of the four primary outcome MAs: work participation and important recovery outcomes. The missed studies did not report data for MAs of functional limitations or pain intensity outcomes, so there were no changes to these MAs. For the former, Table 2 shows the comparison between the results of the original MAs (unadjusted and adjusted) with the results of the MAs of only those studies retrieved by the focused electronic search (“Partial [focused search] meta-analysis [MA]”). In the original MA on work participation outcomes, ten studies were included in the unadjusted MA and twelve were included in the adjusted MA. In the partial MA, those numbers would have dropped to three studies (unadjusted) and four studies (adjusted). In the recovery outcomes MA, the number of included studies would have dropped from three to two (unadjusted) and five to four (adjusted).

**Table 2:** Work participation and important recovery outcome results of Hayden et al.'s [28] original meta-analysis (“Original MA”), the partial meta-analysis including only studies identified using the focused electronic search strategy (i.e., no supplemental searches) (“Partial (focused search) MA”), and the hypothetical broad electronic search strategy (“Partial (broad search) MA”)*

	Number of groups (studies) available for inclusion in meta-analysis (MA)*		Odds ratio (95% CI)	
Outcomes	Unadjusted	Adjusted†	Unadjusted	Adjusted†
Work participation				
Original MA [28]	11 (10)	13 (12)	4.11 (3.46, 4.89)	2.43 (1.64, 3.62)
Partial (focused search) MA	3	4	4.38 (2.48, 7.74)	2.94 (1.86, 4.64)
Partial (broad search) MA	10 (9)	11 (10)	4.05 (3.44, 4.76)	2.72 (2.05, 3.60)
Important recovery				
Original MA	3 (3)	5 (5)	2.40 (1.32, 4.37)	1.89 (1.49, 2.41)
Partial (focused search) MA	2	4	2.83 (1.24, 6.45)	1.88 (1.46, 2.41)
Partial (broad search) MA	No change	No change	No change	No change

* Dichotomous measure of expectations (follow-up closest to 12 months).

† Hayden et al. included study results from the best adjusted model available considering 5 domains of covariates: individual demographics, social support, work factors and environment, psychological factors, and low back pain complaint factors.

MA=meta-analysis; CI=confidence interval.

In the case of the meta-analysis with the largest number of studies available (work participation), the number of participants included in the original MA (n=4,528 in unadjusted MA; n=4,777 in adjusted MA) decreased significantly in the partial MA (n=525 in unadjusted MA; n=731 in adjusted MA), leaving out data on 4,003 potential participants in the unadjusted MA and 4,046 potential participants in the adjusted MA. In our second experiment, limiting to the hypothetical broad electronic search on its own resulted in changes to 1 of the 4 primary outcome MAs: work participation. As shown in Table 2, the number of included studies dropped from 10 to 9 (unadjusted) and 12 to 10 (adjusted). The underlying data for this analysis are found in supplemental Appendix C.

Interpretation of the results did not change in either experiment, as the studies included in the review reported consistently positive associations between expectations and outcomes. In the partial MA, however, confidence intervals (CIs) would have been larger in every case except the adjusted MA of important recovery outcomes. In the work participation outcomes partial MA, the odds ratio (95% CI) would have changed from the original 4.11 (3.46, 4.89) to 4.38 (2.48, 7.74) in the unadjusted MA, and from 2.43 (1.64, 3.62) to 2.94 (1.86, 4.64) in the adjusted MA. In the important recovery outcomes partial MA, the odds ratio (95% CI) would have changed from the original 2.40 (1.32, 4.37) to 2.83 (1.24, 6.45) in the unadjusted MA but remained similar in the adjusted MA (original MA: 1.89 (1.49, 2.41); partial MA: 1.88 (1.46, 2.41)).

### Analysis of prognosis filter performance

The reference standard, drawn from the first stage of screening in the original review, numbered 272 citations. Performance analysis results for each prognosis filter are outlined in Table 3. The Irvin and Combined Hedges + Natural History filters demonstrated similar performances across the board, particularly in sensitivity (90.4% and 90.1%, respectively). The NNR was identical (n=5) for the Irvin, Hedges Optimized, and Inclusive General filters; for the Combined Hedges + Natural History filter, the NNR increased by 1 (n=6).

**Table 3:** Operating characteristics of 4 prognosis filters against the reference standard (n=272), November 2019

Filter	Sensitivity	Precision	NNR	Specificity	Accuracy
Irvin [29]	90.4%	18.3%	5	69.1%	70.6%
Hedges Optimized [9]	73.2%	20.6%	5	78.4%	78.1%
Inclusive General [30]	85.3%	20.2%	5	74.2%	75.0%
Combined Hedges + Natural History [30]	90.1%	17.7%	6	68.1%	69.6%

NNR=number needed to read.

## DISCUSSION

To the best of our knowledge, this methodologic investigation is the first to explore supplementary search methods in the context of searching for prognosis studies. It is also the first effort to directly examine search methods used in a Cochrane review of prognosis studies. This study responds directly to recognized weaknesses in traditional approaches to searching for prognosis studies and aligns with suggestions for supplementary search methods that prominent systematic review methodologists have previously made [17, 26]. Although this is a single case study, the supplementary search methods designed for Hayden et al.'s review can be adapted to suit the purposes of other reviews of prognosis studies.

Each of the individual search components used in the original review retrieved records that were not retrieved by any other component. Leaving out any of the search components (i.e., only running a focused database search) would have resulted in missed studies and would have had an impact on the number of studies included in some of the MAs. Although the observed changes to the MAs' conclusions were not very remarkable in our investigation, it is important to note that the original review consistently found strong, positive associations between the prognostic factor and outcomes of interest; in other words, the review's conclusions were neither ambiguous nor surprising. This made it unlikely that changes to the number of studies that were included would change the outcomes of these MAs. In typical reviews, where the evidence base includes much more uncertainty, it is likely that missing up to 70% of studies would have a far more significant impact on MA conclusions.

Could using a single, broader electronic search have prevented any of these issues? Our exploration of the recall of the focused electronic search strategy that the team used (n=1,332) versus that of a hypothetical broader search (n=15,242) suggests that the broader search, while more sensitive, would have been less feasible to screen. We also found that even the broad search would not have found all studies without also using supplementary search methods. While some review teams may feel more secure running a broader search that leaves fewer stones unturned, we argue that in this case, running a more focused search and putting more comprehensive efforts into supplementary search methods is the more efficient way to locate prognosis studies. Although using prognostic factor terms to focus the search increased the risk of bias in the search results, using supplementary search methods—along with broad inclusion criteria as described by Hayden et al. [28]—helps to mitigate that risk.

The choice of search approaches, however, is highly dependent on the context of the review. While some supplemental search methods (e.g., hand searching, reference searching, forward searching, and consultation of personal files) are possible no matter what the subject matter, some of the supplementary search methods that were investigated in this study would not have been possible if the review had not been in such an active field of research. Reference searching of existing systematic reviews is only possible if there have been previous attempts to synthesize the evidence on a topic. Similarly, identifying relevant prognostic factor measures can be more straightforward if the research team is already aware of literature on a topic. Thus, the supplementary search methods we investigated may be best applied to large reviews of well-established prognosis topics, and those conducting reviews in newer research areas may be better advised to run a broad electronic search.

The large size of Hayden et al.'s review affects our analysis in other ways. The difference in recall between a focused and broad electronic search strategy might not be so stark in a smaller review, potentially making supplementary search methods less productive. However, even in smaller reviews, supplementary searching remains necessary to retrieve all relevant studies, at least until prognosis studies become more findable (e.g., by applying better indexing and using more consistent prognosis terminology) or until databases improve controlled vocabulary and synonymy for prognosis concepts.

Our prognosis filter analysis found that the filter that the original review team used (the Irvin filter [29]) had the highest sensitivity (90.4%) of any of the other filters we tested and had an identical or near identical NNR (n=5). Even though the Irvin filter's sensitivity was the best in our test group, it was still not sensitive enough to retrieve all of the studies in our reference standard. This again highlights the importance of not relying solely on a prognosis filter to locate prognosis studies. Furthermore, our findings suggest it may be unreasonable for searchers to expect that a single, ideal prognosis filter could possibly be created to apply in all prognosis searching contexts. However, our ad hoc filter testing is not a substitute for systematically testing every prognosis filter that has been validated in the literature—our team is aware of one such effort currently being undertaken [5]—nor can it take the place of a formal filter validation study.

It remains unknown whether the focused electronic search strategy used in this example is the “ideal” strategy. While the comprehensiveness of a search strategy can be difficult to absolutely ascertain, the team may have benefitted from using a more iterative, “pearl growing,” search development approach to harvest additional relevant search terms [33]. Iterative search development is particularly important in the prognostic factor context, in which terminology can vary from topic to topic. Using a more iterative approach might have increased the sensitivity of the focused search without adding greatly to recall and could have been very effective in this case, where a large body of evidence—including previous syntheses—was already in existence.

Our analysis was done retrospectively, and it was impossible to make firm time estimates for each of the search components used. Future research on prognosis search methods could conduct a more rigorous, real-time comparison of traditional versus supplementary search methods. Future research could also explore iterative search methods in a prognosis context. Finally, methodologic investigations of reviews of prognosis on topics other than low back pain would add greatly to the field of prognosis search methods research.

## Data Availability

All data associated with this article are available in supplemental Appendix D.

## SUPPLEMENTAL FILES

**Appendix A:** Focused Ovid MEDLINE search strategy used in Hayden et al.'s Cochrane review on the association between recovery expectations and disability outcomes in adults with low back pain

**Appendix B:** Supplemental tables

**Appendix C:** Data used in meta-analyses of association of expectations with outcomes

**Appendix D:** Associated data
