# Supplementary material for: Search methods for prognostic factor systematic reviews: a methodologic investigation
Source: J Med Libr Assoc. 2021 Jan 1;109(1):23–32. doi: 10.5195/jmla.2021.939 (PMC7772979; doi:10.5195/jmla.2021.939)
Supplement: Supplementary file 1 — Appendix A: Focused Ovid MEDLINE search strategy used in Hayden et al.'s Cochrane review on the association between recovery expectations and disability outcomes in adults with low back pain [file jmla-109-1-23-s01.pdf]

JMLA Journal of the Medical Library Association

SC

Supplemental content to
J Med Libr Assoc. Jan;109(1):dx.doi.org/10.5195/jmla.2021.939
www.jmla.mlanet.org
© Boulos, Ogilvie, Hayden 2021

Search methods for prognostic factor systematic reviews: a methodologic investigation

Leah Boulos; Rachel Ogilvie; Jill A. Hayden

APPENDIX A

Focused Ovid MEDLINE search strategy used in Hayden et al.'s Cochrane review on the association between recovery expectations and disability outcomes in adults with low back pain

1. dorsalgia.ti,ab.
2. exp Back Pain/
3. back pain.ti,ab.
4. backache.ti,ab.
5. back ache.ti,ab.
6. lumb* pain.ti,ab.
7. coccyx.ti,ab.
8. coccydynia.ti,ab.
9. sciatica.ti,ab.
10. exp sciatic neuropathy/
11. sciatic neuropathy.ti,ab.
12. spondylosis.ti,ab.
13. lumbago.ti,ab.
14. back disorder$.ti,ab.
15. back injur$.ti,ab.
16. or/1-15
17. expectancy.tw.
18. expectation*.tw.
19. exp Attitude to Health/
20. Health Knowledge, Attitudes, Practice/
21. self efficacy/
22. self efficacy.tw.
23. illness belief*.tw.
24. ((disab* or self* or injur*) adj3 percept*).tw.
25. expectation*.tw.
26. (outcome adj3 expect*).tw.
27. (questionnaire* adj3 (belief* or hope* or perceive* or expect* or desire* or percept* or likelihood or likely or anticipat* or want* or certainty or self-efficacy)).tw.
28. (recovery* adj3 (belief* or hope* or perceive* or expect* or desire* or percept* or likelehood or likely or anticipat* or want* or certainty or self-efficacy)).tw.
29. (measure* adj3 (belief* or hope* or perceive* or expect* or desire* or percept* or likelihood or likely or anticipat* or want* or certainty or self-efficacy)).tw.

JMLA Journal of the Medical Library Association

SC
Supplemental content to
J Med Libr Assoc. Jan;109(1):dx.doi.org/10.5195/jmla.2021.939
www.jmla.mlanet.org
© Boulos, Ogilvie, Hayden 2021

30. or/17-29
31. Cohort Studies/
32. incidence.tw.
33. Mortality/
34. Follow-Up Studies/
35. prognos*.tw.
36. predict*.tw.
37. course.tw.
38. Survival Analysis/
39. or/31-38
40. 16 and 30 and 3
