# Supplementary material for: Search methods for prognostic factor systematic reviews: a methodologic investigation
Source: J Med Libr Assoc. 2021 Jan 1;109(1):23–32. doi: 10.5195/jmla.2021.939 (PMC7772979; doi:10.5195/jmla.2021.939)
Supplement: Supplementary file 2 — Appendix B: Supplemental tables [file jmla-109-1-23-s02.pdf]

JMLA Journal of the Medical Library Association

SC
Supplemental content to
J Med Libr Assoc. Jan;109(1):dx.doi.org/10.5195/jmla.2021.939
www.jmla.mlanet.org
© Boulos, Ogilvie, Hayden 2021

Search methods for prognostic factor systematic reviews: a methodologic investigation

Leah Boulos; Rachel Ogilvie; Jill A. Hayden

APPENDIX B

Supplemental tables

Supplemental Table 1 Search methods of origin for each study included in Hayden et al.'s Cochrane review [28] that is indexed in Ovid MEDLINE (n=58 out of 60)*

Study ID (author/year)	Focused electronic database search for primary studies	Systematic review reference search	Forward search of publications citing known prognostic factor measures	Hand searching, reference searching, and personal files	Broad electronic database search for primary studies (hypothetical)†
Beneciuk 2017				X	X
Besen 2015	X	X			X
Bishop 2015				X	
Butler 2007		X			
Carriere 2015				X	
Demmelmaier 2010	X	X			X
Dionne 1997				X	X
Downie 2016	X				X
Du Bois 2008				X	X
Enthoven 2006	X	X			X
Enthoven 2016	X		X		X
Foster 2008	X	X			X
George 2010	X				X
Gervais 1991	X	X			X
Glattacker 2013	X	X			X
Goldstein 2002	X	X			X
Gross 2010	X	X			X
Grotle 2006				X	X
Haas 2014				X	

JMLA Journal of the Medical Library Association

SC
Supplemental content to
J Med Libr Assoc. Jan;109(1):dx.doi.org/10.5195/jmla.2021.939
www.jmla.mlanet.org
© Boulos, Ogilvie, Hayden 2021

Study ID (author/year)	Focused electronic database search for primary studies	Systematic review reference search	Forward search of publications citing known prognostic factor measures	Hand searching, reference searching, and personal files	Broad electronic database search for primary studies (hypothetical)†
Hagen 2005		X			X
Haldorsen 1998				X	X
Hazard 1996				X	X
Henschke 2008				X	X
Heymans 2006	X				X
Hildebrandt 1997	X	X			X
Jellema 2002	X	X			X
Jensen 2000	X	X			X
Jensen 2013	X	X			X
Karjalainen 2003	X				X
Kongsted 2014	X				X
Leboeuf-Yde 2004			X		X
Lindell 2010	X	X			X
Macedo 2014	X				X
Magnussen 2007	X	X			X
Michaelson 2004	X				X
Myers 2008	X	X			X
Niemistö 2004				X	X
Opsahl 2016				X	X
Opsommer 2017	X				X
Petersen 2007	X	X			X
Rasmussen-Barr 2012	X	X			X
Reeser 2001			X		
Reiso 2003		X			X
Reme 2009	X		X		X

JMLA Journal of the Medical Library Association

SC
Supplemental content to
J Med Libr Assoc. Jan;109(1):dx.doi.org/10.5195/jmla.2021.939
www.jmla.mlanet.org
© Boulos, Ogilvie, Hayden 2021

Study ID (author/year)	Focused electronic database search for primary studies	Systematic review reference search	Forward search of publications citing known prognostic factor measures	Hand searching, reference searching, and personal files	Broad electronic database search for primary studies (hypothetical)†
Rundell 2017				X	X
Sandstrom 1986	X				X
Schultz 2004	X				X
Shaw 2009	X				X
Sherman 2009	X				X
Steenstra 2005				X	X
Tran 2015	X				X
Truchon 2012	X				X
Turner 2008				X	X
Underwood 2007				X	X
Van Hooff 2014				X	X
van Wijk 2008	X				X
Yelland 2006	X				X
Young Casey 2008				X	X
Total	35	20	4	18	53

* Search recall and search filter analyses in this methodologic investigation were limited to Ovid MEDLINE. Incidentally, the two studies not located in Ovid MEDLINE were not included in the review's meta-analyses.

† A broad electronic database search for primary studies was not undertaken as part of the review's actual search methods; this is a hypothetical search to show the difference in recall.

Journal of the Medical Library Association

Supplemental content to
J Med Libr Assoc. Jan;109(1):dx.doi.org/10.5195/jmla.2021.939
www.jmla.mlanet.org
© Boulos, Ogilvie, Hayden 2021

Supplemental Table 2 References for studies included in supplemental Table 1

Study ID (author/year)	Citations
Beneciuk 2017	Beneciuk JM, Hill JC, Campbell P, Afolabi E, George SZ, Dunn KM, Foster NE. Identifying treatment effect modifiers in the STarT Back trial: a secondary analysis. J Pain. 2017 Jan;18(1):54–65.
Besen 2015	Besen E, Young AE, Shaw WS. Returning to work following low back pain: towards a model of individual psychosocial factors. J Occup Rehabil. 2015 Mar;25(1):25–37.
Bishop 2015	Bishop FL, Yardley l, Prescott P, Cooper C, Little P, Lewith GT. Psychological covariates of longitudinal changes in back-related disability in patients undergoing acupuncture. Clin J Pain. 2015 Mar;31(3):254–64.
Butler 2007	Butler RJ, Johnson WG, Côté P. It pays to be nice: employer-worker relationships and the management of back pain claims. J Occup Environ Med. 2007 Feb;49(2):214–5.
Carriere 2015	Carriere J, Thibault P, Sullivan M. The mediating role of recovery expectancies on the relation between depression and return-to-work. J Occup Rehabil. 2015 Jun;25(2):348–56.
Demmelmaier 2010	Demmelmaier I, Asenlof P, Lindberg P, Denison E. Biopsychosocial predictors of pain, disability, health care consumption, and sick leave in first-episode and long-term back pain: a longitudinal study in the general population. Int J Behav Med. 2010 Jun;17(2):79–89.
Dionne 1997	Dionne CE, Koepsell TD, Korff M, Deyo RA, Barlow WE, Checkoway H. Predicting long-term functional limitations among back pain patients in primary care settings. J Clin Epidemiol. 1997 Jan;50(1):31-43.
Downie 2016	Downie AS, Hancock MJ, Rzewuska M, Williams CM, Lin CW, Maher CG. Trajectories of acute low back pain: a latent class growth analysis. Pain. 2016 Jan;157(1):225–34.
Du Bois 2008	Du Bois M, Donceel P. A screening questionnaire to predict no return to work within 3 months for low back pain claimants. Eur Spine J. 2008 Mar;17(3):380–5.
Enthoven 2006	Enthoven P, Skargren E, Carstensen J, Oberg B. Predictive factors for 1-year and 5-year outcome for disability in a working population of patients with low back pain treated in primary care. Pain. 2006 May;122(1–2):137–44.
Enthoven 2016	Enthoven WT, Koes BW, Bierma-Zeinstra SM, Bueving HJ, Bohnen AM, Peul WC, van Tulder MW, Berger MY, Luijsterburg PAJ. Defining trajectories in older adults with back pain presenting in general practice. Age Ageing. 2016 Nov;45(6):878–83.
Foster 2008	Foster NE, Bishop A, Thomas E, Main C, Horne R, Weinman J, Hay E. Illness perceptions of low back pain patients in primary care: what are they, do they change and are they associated with outcome? Pain. 2008 May;136(1–2):177–87.
George 2010	George SZ, Robinson ME. Preference, expectation, and satisfaction in a clinical trial of behavioral interventions for acute and sub-acute low back pain. J Pain. 2010 Nov;11(11):1074–82.

SC
Supplemental content to
J Med Libr Assoc. Jan;109(1):dx.doi.org/10.5195/jmla.2021.939
www.jmla.mlanet.org
© Boulos, Ogilvie, Hayden 2021

Study ID (author/year)	Citations
Gervais 1991	Gervais S, Dupuis G, Veronneau F, Bergeron Y, Millette D, Avard J. Predictive model to determine cost/benefit of early detection and intervention in occupational low back pain. J Occup Rehabil. 1991 Jun;1(2):113–31.
Glattacker 2013	Glattacker M, Heyduck K, Meffert C. Illness beliefs and treatment beliefs as predictors of short-term and medium-term outcome in chronic back pain. J Rehabil Med. 2013 Mar;45(3):268–76.
Goldstein 2002	Goldstein MS, Morgenstern H, Hurwitz EL, Yu F. The impact of treatment confidence on pain and related disability among patients with low-back pain: results from the University of California, Los Angeles, low-back pain study. Spine J. 2002 Nov–Dec;2(6):391–9.
Gross 2010	Gross DP, Battie MC. Recovery expectations predict recovery in workers with back pain but not other musculoskeletal conditions. J Spinal Disorders Tech. 2010 Oct;23(7):451–6.
Grotle 2006	Grotle M, Vøllestad NK, Brox JI. Screening for yellow flags in first-time acute low back pain: reliability and validity of a Norwegian version of the acute low back pain screening questionnaire. Clin J Pain. 2006 Jun;22(5):458–67.
Haas 2014	Haas M, Vavrek D, Neradilek MB, Polissar N. A path analysis of the effects of the doctor-patient encounter and expectancy in an open-label randomized trial of spinal manipulation for the care of low back pain. BMC Complement Alternative Med. 2014 Jan 13;14:16.
Hagen 2005	Hagen EM, Svensen E, Ericksen HR. Predictors and modifiers of treatment effect influencing sick leave in subacute low back pain patients. Spine (Phila Pa 1976). 2005 Dec 15;30(24):2717–23.
Haldorsen 1998	Haldorsen EM, Kronholm K, Skouen JS, Ursin H. Predictors for outcome of a multi-modal cognitive behavioural treatment program for low back pain patients - a 12-month follow-up study. Eur J Pain. 1998;2(4):293–307.
Hazard 1996	Hazard RG, Haugh LD, Reid S, Preble JB, MacDonald L. Early prediction of chronic disability after occupational low back injury. Spine (Phila Pa 1976). 1996 Apr 15;21(8):945–51.
Henschke 2008	Henschke N, Maher CG, Refshauge KM, Herbert RD, Cumming RG, Bleasel J, York J, Das A, McAuley JH. Prognosis in patients with recent onset low back pain in Australian primary care: inception cohort study. BMJ. 2008 Jul 7;337(7662):a171.
Heymans 2006	Heymans MW, Vet HC, Knol DL, Bongers PM, Koes BW, Mechelen W. Workers' beliefs and expectations affect return to work over 12 months. J Occup Rehabil. 2006 Dec;16(4):685–95.
Hildebrandt 1997	Hildebrandt J, Pfingsten M, Saur P, Jansen J. Prediction of success from a multidisciplinary treatment program for chronic low back pain. Spine (Phila Pa 1976). 1997 May 1;22(9):990–1001.

SC
Supplemental content to
J Med Libr Assoc. Jan;109(1):dx.doi.org/10.5195/jmla.2021.939
www.jmla.mlanet.org
© Boulos, Ogilvie, Hayden 2021

Study ID (author/year)	Citations
Jellema 2002	Jellema P, Bierma-Zeinstra SM, Poppel MN, Bernsen RM, Koes BW. Feasibility of lumbar supports for home care workers with low back pain. Occup Med (London). 2002 Sep;52(6):317–23.
Jensen 2000	Jensen IB, Bodin L, Ljungqvist T, Gunnar Bergström K, Nygren A. Assessing the needs of patients in pain: a matter of opinion? Spine (Phila Pa 1976). 2000 Nov 1;25(21):2816–23.
Jensen 2013	Jensen OK, Stengaard-Pedersen K, Jensen C, Nielsen CV. Prediction model for unsuccessful return to work after hospital-based intervention in low back pain patients. BMC Musculoskelet Disord. 2013 Apr 19;14:140.
Karjalainen 2003	Karjalainen K, Malmivaara A, Mutanen P, Pohjolainen T, Roine R, Hurri H. Outcome determinants of subacute low back pain. Spine (Phila Pa 1976). 2003 Dec 1;28(23):2634–40.
Kongsted 2014	Kongsted A, Vach W, Axø M, Bech RN, Hestbaek L. Expectation of recovery from low back pain: a longitudinal cohort study investigating patient characteristics related to expectations and the association between expectations and 3-month outcome. Spine (Phila Pa 1976). 2014 Jan 1;39(1):81–90.
Leboeuf-Yde 2004	Leboeuf-Yde C, Gronstvedt A, Borge JA, Lothe J, Magnesen E, Nilsson O, Røsok G, Stig LC, Larsen K. The Nordic Back Pain Subpopulation Program: demographic and clinical predictors for outcome in patients receiving chiropractic treatment for persistent low-back pain. J Manip Physiol Ther. 2004 Oct;27(8):493–502.
Lindell 2010	Lindell O, Johansson SE, Strender LE. Predictors of stable return-to-work in non-acute, non-specific spinal pain: low total prior sick-listing, high self prediction and young age. a two-year prospective cohort study. BMC Family Pract. 2010 Jul 20;11:53.
Macedo 2014	Macedo LG, Maher CG, Hancock MJ, Kamper SJ, McAuley JH, Stanton TR, Stafford R, Hodges PW. Predicting response to motor control exercises and graded activity for patients with low back pain: preplanned secondary analysis of a randomized controlled trial. Phys Ther. 2014 Nov;94(11):1543–54.
Magnussen 2007	Magnussen L, Strand LI, Skouen JS, Eriksen HR. Motivating disability pensioners with back pain to return to work--a randomized controlled trial. J Rehabil Med. 2007 Jan;39(1):81–7.
Michaelson 2004	Michaelson P, Sjölander P, Johansson H. Factors predicting pain reduction in chronic back and neck pain after multimodal treatment. Clin J Pain. 2004 Nov–Dec;20(6):447–54.
Myers 2008	Myers SS, Phillips RS, Davis RB, Cherkin DC, Legedza A, Kaptchuk TJ, Hrbek A, Buring JE, Post D, Connelly MT, Eisenberg DM. Patient expectations as predictors of outcome in patients with acute low back pain. J Gen Intern Med. 2008 Feb;23(2):148–53.

SC
Supplemental content to
J Med Libr Assoc. Jan;109(1):dx.doi.org/10.5195/jmla.2021.939
www.jmla.mlanet.org
© Boulos, Ogilvie, Hayden 2021

Study ID (author/year)	Citations
Niemistö 2004	Niemistö L, Sarna S, Lahtinen-Suopanki T, Lindgren KA, Hurri H. Predictive factors for 1-year outcome of chronic low back pain following manipulation, stabilizing exercises, and physician consultation or physician consultation alone. J Rehabil Med. 2004 May;36(3):104–9.
Opsahl 2016	Opsahl J, Eriksen HR, Tveito TH. Do expectancies of return to work and job satisfaction predict actual return to work in workers with long lasting LBP? BMC Musculoskelet Disord. 2016 Nov 17;17(1):481.
Opsommer 2017	Opsommer E, Rivier G, Crombez G, Hilfiker R. The predictive value of subsets of the Örebro musculoskeletal pain screening questionnaire for return to work in chronic low back pain. Eur J Phys Rehabil Med. 2017 Jun;53(3):359–65.
Petersen 2007	Petersen T, Larsen K, Jacobsen S. One-year follow-up comparison of the effectiveness of McKenzie treatment and strengthening training for patients with chronic low back pain: outcome and prognostic factors. Spine (Phila Pa 1976). 2007 Dec 15;32(26):2948–56.
Rasmussen-Barr 2012	Rasmussen-Barr E, Campello M, Arvidsson I, Nilsson-Wikmar L, Ang BO. Factors predicting clinical outcome 12 and 36 months after an exercise intervention for recurrent low-back pain. Disabil Rehabil. 2012;34(2):136–44.
Reeser 2001	Reeser JC, Wiegmann SM, Hoover N, Oldridge N, Phillips D, Bjelland T, Scarpinato L, Treacy W, Helstad CP, Stoll J. Treatment of acute low back pain in Wisconsin: results of the State Medical Society's medical outcomes research project. Wis Med J. 2001;100(8):35–42.
Reiso 2003	Reiso H, Nygård JF, Jorgensen GS, Holanger R, Soldal D, Bruusgaard D. Back to work: predictors of return to work among patients with back disorders certified as sick: a two-year follow-up study. Spine (Phila Pa 1976). 2003 Jul 1;28(13):1468–73; discussion 1473–4.
Reme 2009	Reme SE, Hagen EM, Eriksen HR. Expectations, perceptions, and physiotherapy predict prolonged sick leave in subacute low back pain. BMC Musculoskel Disord. 2009 Nov 13;10:139.
Rundell 2017	Rundell SD, Sherman KJ, Heagerty PJ, Mock CN, Dettori NJ, Comstock BA, Avins AL, Nedeljkovic SS, Nerenz DR, Jarvik JG. Predictors of persistent disability and back pain in older adults with a new episode of care for back pain. Pain Med. 2017 Jun 1;18(6):1049–62.
Sandstrom 1986	Sandstrom J, Esbjörnsson E. Return to work after rehabilitation. the significance of the patient's own prediction. Scand J Rehabil Med. 1986;18(1):29–33.
Schultz 2004	Schultz IZ, Crook J, Meloche GR, Berkowitz J, Milner R, Zuberbier OA, Meloche W. Psychosocial factors predictive of occupational low back disability: towards development of a return-to-work model. Pain. 2004 Jan;107(1–2):77–85.
Shaw 2009	Shaw WS, Pransky G, Winters T. The back disability risk questionnaire for work-related, acute back pain: prediction of unresolved problems at 3-month follow-up. J Occup Environ Med. 2009 Feb;51(2):185–94.

SC
Supplemental content to
J Med Libr Assoc. Jan;109(1):dx.doi.org/10.5195/jmla.2021.939
www.jmla.mlanet.org
© Boulos, Ogilvie, Hayden 2021

Study ID (author/year)	Citations
Sherman 2009	Sherman KJ, Cherkin DC, Ichikawa L, Avins AL, Barlow WE, Khalsa PS, Deyo RA. Characteristics of patients with chronic back pain who benefit from acupuncture. BMC Musculoskel Disord. 2009 Sep 21;10:114.
Steenstra 2005	Steenstra IA, Koopman FS, Knol DL, Kat E, Bongers PM, de Vet HCW, van Mechelen W. Prognostic factors for duration of sick leave due to low-back pain in Dutch health care professionals. J Occup Rehabil. 2005 Dec;15(4):591–605.
Tran 2015	Tran HH, Weinberg J, Sherman KJ, Saper RB. Preference and expectation for treatment assignment in a randomized controlled trial of once- vs twice-weekly yoga for chronic low back pain. Glob Advanc Health Med. 2015 Jan;4(1):34–9.
Truchon 2012	Truchon M, Schmouth M, Fillion AL, Rossignol M, Durand M. Absenteeism screening questionnaire (ASQ): a new tool for predicting long-term absenteeism among workers with low back pain. J Occup Rehabil. 2012 Mar;22(1):27–50.
Turner 2008	Turner JA, Franklin G, Fulton-Kehoe D, Sheppard L, Stover B, Wu R, Gluck JV, Wickizer TM. ISSLS prize winner: early predictors of chronic work disability: a prospective, population-based study of workers with back injuries. Spine (Phila Pa 1976). 2008 Dec 1;33(25):2809–18.
Underwood 2007	Underwood MR, Morton, Farrin A; UK BEAM Trial Team. Do baseline characteristics predict response to treatment for low back pain? secondary analysis of the UK BEAM dataset. Rheumatology (Oxford). 2007 Aug;46(8):1297–302.
van Hooff 2014	van Hooff ML, Spruit M, O'Dowd JK, van Lankveld W, Fairbank JC, van Limbeek J. Predictive factors for successful clinical outcome 1 year after an intensive combined physical and psychological programme for chronic low back pain. Eur Spine J. 2014 Jan;23(1):102–12.
van Wijk 2008	van Wijk RMAW, Geurts JWM, Lousberg R, Wynne HJ, Hammink E, Knape JTA, Groen GJ. Psychological predictors of substantial pain reduction after minimally invasive radiofrequency and injection treatments for chronic low back pain. Pain Med. 2008 Mar;9(2):212–21.
Yelland 2006	Yelland MJ, Schluter PJ. Defining worthwhile and desired responses to treatment of chronic low back pain. Pain Med. 2006 Jan–Feb;7(1):38–45.
Young Casey 2008	Young Casey C, Greenberg MA, Nicassio PM, Harpin RE, Hubbard D. Transition from acute to chronic pain and disability: a model including cognitive, affective, and trauma factors. Pain. 2008 Jan;134(1–2):69–79.
