# Supplementary material for: Search methods for prognostic factor systematic reviews: a methodologic investigation
Source: J Med Libr Assoc. 2021 Jan 1;109(1):23–32. doi: 10.5195/jmla.2021.939 (PMC7772979; doi:10.5195/jmla.2021.939)
Supplement: Supplementary file 3 — Appendix C: Data used in meta-analyses of association of expectations with outcomes [file jmla-109-1-23-s03.pdf]

JMLA Journal of the Medical Library Association

SC
Supplemental content to
J Med Libr Assoc. Jan;109(1):dx.doi.org/10.5195/jmla.2021.939
www.jmla.mlanet.org
© Boulos, Ogilvie, Hayden 2021

Search methods for prognostic factor systematic reviews: a methodologic investigation

Leah Boulos; Rachel Ogilvie; Jill A. Hayden

APPENDIX C

Data used in meta-analyses of association of expectations with outcomes

Source: Secondary analysis of data from Hayden et al. [28]

Supplemental Table 3 Data used in meta-analyses of association of expectations with outcomes with all included studies

MA name, study ID	Effect estimate	SE	Confidence interval (CI) start	CI end
Return to work (RTW) outcome, dichotomous measure of expectations, ALL STUDIES				
Unadjusted results	4.11		3.46	4.89
Carriere 2015	13.60	0.79	2.89	63.97
Du Bois 2008	5.21	0.39	2.42	11.18
Hagen 2005	2.29	0.25	1.40	3.74
Jensen 2013	3.25	0.38	1.55	6.85
Lindell 2010	6.42	0.61	1.94	21.23
Magnussen 2007	6.89	0.66	1.89	25.12
Opsahl 2016a (women)*	4.81	0.34	2.47	9.36
Opsahl 2016b (men)*	5.37	0.33	2.81	10.24
Reiso 2003	3.97	0.56	1.33	11.91
Steenstra 2005	4.06	0.14	3.08	5.34
Turner 2008	4.31	0.16	3.15	5.89
Adjusted results	2.43		1.64	3.62
Butler 2007	1.15	0.41	0.52	2.57
Carriere 2015	1.04	0.01	1.02	1.06
Du Bois 2008	4.62	0.41	2.07	10.31
Hagen 2005	1.80	0.30	1.00	3.25
Jensen 2013	2.94	0.33	1.54	5.62
Lindell 2010	5.21	0.62	1.54	17.55
Opsahl 2016a (women)*	4.18	0.40	1.91	9.15
Opsahl 2016b (men)*	3.35	0.38	1.59	7.06
Petersen 2007	6.49	0.78	1.41	29.93
Reiso 2003	3.22	0.29	1.83	5.69
Reme 2009	1.90	0.38	0.90	3.99
Steenstra 2005	2.83	0.17	2.03	3.95
Turner 2008	1.30	0.21	0.86	1.96

JMLA Journal of the Medical Library Association

SC
Supplemental content to
J Med Libr Assoc. Jan;109(1):dx.doi.org/10.5195/jmla.2021.939
www.jmla.mlanet.org
© Boulos, Ogilvie, Hayden 2021

MA name, study ID	Effect estimate	SE	Confidence interval (CI) start	CI end
Important recovery outcome, dichotomous measure of expectations, ALL STUDIES				
Unadjusted results	2.40		1.32	4.37
Niemistö 2004	1.75	0.34	0.90	3.41
Rasmussen-Barr 2012	1.60	0.56	0.53	4.80
Shaw 2009	3.86	0.29	2.18	6.81
Adjusted results	1.89		1.49	2.41
Enthoven 2006	1.19	0.54	0.41	3.42
Foster 2008	1.84	0.15	1.37	2.47
Niemistö 2004	2.12	0.44	0.89	5.01
Petersen 2007	1.80	0.43	0.78	4.19
Shaw 2009	2.69	0.37	1.30	5.56

* Opsahl 2016 presented data for women (n=286) and men (n=283) separately.

Supplemental Table 4 Data used in meta-analyses of association of expectations with outcomes with studies excluded that were not identified in the focused search ("Partial MA")

MA name, study ID	Effect estimate	SE	CI start	CI end
RTW outcome, dichotomous measure of expectations, partial MA: excluding studies not identified in focused search				
Unadjusted results	4.38		2.48	7.74
Jensen 2013	3.25	0.38	1.55	6.85
Lindell 2010	6.42	0.61	1.94	21.23
Magnussen 2007	6.89	0.66	1.89	25.12
Adjusted results	2.94		1.86	4.64
Jensen 2013	2.94	0.33	1.54	5.62
Lindell 2010	5.21	0.62	1.54	17.55
Petersen 2007	6.49	0.78	1.41	29.93
Reme 2009	1.90	0.38	0.90	3.99
Important recovery outcome, dichotomous measure of expectations, partial MA: excluding studies not identified in focused search				
Unadjusted results	2.83		1.24	6.45
Rasmussen-Barr 2012	1.60	0.56	0.53	4.80
Shaw 2009	3.86	0.29	2.18	6.81
Adjusted results	1.88		1.46	2.41
Enthoven 2006	1.19	0.54	0.41	3.42
Foster 2008	1.84	0.15	1.37	2.47
Petersen 2007	1.80	0.43	0.78	4.19
Shaw 2009	2.69	0.37	1.30	5.56

SC
Supplemental content to
J Med Libr Assoc. Jan;109(1):dx.doi.org/10.5195/jmla.2021.939
www.jmla.mlanet.org
© Boulos, Ogilvie, Hayden 2021

Supplemental Table 5 Data used in meta-analyses of association of expectations with outcomes with studies excluded that were not identified in the broad search ("Broad MA")

MA name, study ID	Effect estimate	SE	CI start	CI end
RTW outcome, dichotomous measure of expectations, Broad MA: excluding studies not identified in broad search				
Unadjusted results	4.05		3.44	4.76
Du Bois 2008	5.21	0.39	2.42	11.18
Hagen 2005	2.29	0.25	1.40	3.74
Jensen 2013	3.25	0.38	1.55	6.85
Lindell 2010	6.42	0.61	1.94	21.23
Magnussen 2007	6.89	0.66	1.89	25.12
Opsahl 2016a (women)*	4.81	0.34	2.47	9.36
Opsahl 2016b (men)*	5.37	0.33	2.81	10.24
Reiso 2003	3.97	0.56	1.33	11.91
Steenstra 2005	4.06	0.14	3.08	5.34
Turner 2008	4.31	0.16	3.15	5.89
Adjusted results	2.72		2.05	3.60
Du Bois 2008	4.62	0.41	2.07	10.31
Hagen 2005	1.80	0.30	1.00	3.25
Jensen 2013	2.94	0.33	1.54	5.62
Lindell 2010	5.21	0.62	1.54	17.55
Opsahl 2016a (women)*	4.18	0.40	1.91	9.15
Opsahl 2016b (men)*	3.35	0.38	1.59	7.06
Petersen 2007	6.49	0.78	1.41	29.93
Reiso 2003	3.22	0.29	1.83	5.69
Reme 2009	1.90	0.38	0.90	3.99
Steenstra 2005	2.83	0.17	2.03	3.95
Turner 2008	1.30	0.21	0.86	1.96
Important recovery outcome, dichotomous measure of expectations, Broad MA: excluding studies not identified in broad search				
Unadjusted results	2.40		1.32	4.37
Niemistö 2004	1.75	0.34	0.90	3.41
Rasmussen-Barr 2012	1.60	0.56	0.53	4.80
Shaw 2009	3.86	0.29	2.18	6.81
Adjusted results	1.89		1.49	2.41
Enthoven 2006	1.19	0.54	0.41	3.42
Foster 2008	1.84	0.15	1.37	2.47
Niemistö 2004	2.12	0.44	0.89	5.01
Petersen 2007	1.80	0.43	0.78	4.19
Shaw 2009	2.69	0.37	1.30	5.56

* Opsahl 2016 presented data for women (n=286) and men (n=283) separately.
